# Comparison of different symmetry indices for the quantification of dynamic joint angles

**DOI:** 10.1186/s13102-021-00355-4

**Published:** 2021-10-19

**Authors:** Hannah Lena Siebers, Waleed Alrawashdeh, Marcel Betsch, Filippo Migliorini, Frank Hildebrand, Jörg Eschweiler

**Affiliations:** 1 Department of Orthopaedics, Trauma and Reconstructive Surgery, University Hospital RWTH Aachen, Pauwelsstr. 30, 52074 Aachen, Germany; 2 Department of Orthopaedics and Trauma Surgery, University Medical Center Mannheim of the University Heidelberg, Mannheim, Germany

**Keywords:** Gait analysis, Stair climbing, Gait symmetry, IMU

## Abstract

**Background:**

Symmetry is a sign of physiological and healthy movements, as pathologies are often described by increased asymmetries. Nevertheless, based on precisely measured data, even healthy individuals will show small asymmetries in their movements. However, so far there do not exist commonly accepted methods and reference values for gait symmetry in a healthy collective. Therefore, a comparison and presentation of reference values calculated by 3 different methods of symmetry indices for lower limb joint angles during walking, ascending, and descending stairs were shown.

**Methods:**

Thirty-five healthy participants were analyzed during walking, ascending, and descending stairs with the help of the inertial measurement system MyoMotion. Using the normalized symmetry index (SI_norm_), the symmetry index (SI) as the integral of the symmetry function, and another normalized symmetry index (NSI), the symmetry of joint angles was evaluated. For statistical evaluation of differences, repeated measurement models and Bland–Altman-Plots were used.

**Results:**

Apart from a bias between the symmetry indices, they were comparable in the predefined limits of 5%. For all parameters, significantly higher asymmetry was found for ankle dorsi/-plantarflexion, compared with the hip and knee flexion. Moreover, the interaction effect of the joint and movement factors was significant, with an increased asymmetry of the hip and knee during descending stairs greater than while ascending stairs or walking, but a reduced symmetry of the ankle during walking when compared to descending. The movement only showed significant effects when analyzing the SI_norm_**.**

**Conclusion:**

Even for healthy individuals, small asymmetries of movements were found and presented as reference values using 3 different symmetry indices for dynamic lower limb joint angles during 3 different movements. For the quantification of symmetrical movements differences between the joints, movements, and especially their interaction, are necessary to be taken into account. Moreover, a bias between the methods should be noted. The potential for each presented symmetry index to identify pathological movements or track a rehabilitation process was shown but has to be proven in further research.

*Trial registration*: DRKS00025878.

## Introduction

As far as the musculoskeletal system is concerned, humans are largely symmetrical beings. Assuming this symmetry, in medicine, the comparison of affected and non-affected sides is a popular and proven tool in the diagnostics and treatment of musculoskeletal disorders [1–4]. Also, during a range of movements, like walking, running, stair climbing, and cycling, a symmetrical execution is described as a sign of physiologically healthy movement, and therefore, a treatment goal [5]. Due to the recent developments in measurement technology and software in the last decades, symmetry analysis is gaining more and more interest [5–7]. Objectively measured values can be detected continuously [8], a possible offset can be corrected and data from different time points can be compared [9]. Due to differences in motion data and measurement systems along with large amounts of data, different methods for symmetry analysis have been described [10, 11]. Up to date, there is no generally accepted standard for assessing symmetry. This fact makes it difficult, to compare studies and establish criteria to guide clinical decision-making.

The symmetry index (SI, Eq. 1) is one of the most frequently used parameters, which was also described as the clinically most sensitive parameter in the investigation of spatiotemporal gait parameters [11].

1 $${\text{SI}} = \frac{{{\text{X}}_{{\text{R}}} - {\text{X}}_{{\text{L}}} }}{{0.5* \left( {{\text{X}}_{{\text{R}}} + {\text{X}}_{{\text{L}}} } \right)}} \cdot 100\%$$

However, the SI is not suitable for the analysis of joint angles over a complete gait cycle, since artificial extreme values occur in the region of zero-crossings [12, 13]. Based on literature research, three methods were identified that allow for the calculation and presentation of a symmetry parameter over a complete gait cycle [9, 10, 12]. Compared to other methods, analyzing only specific parameters like the range of motion/maximum joint angle, which only takes into account a minimum of the measured information [10], the complete gait cycle can be analyzed. Moreover, presenting symmetry parameters over the complete gait cycle enables the identification and interpretation of symmetrical and asymmetrical parts during the gait cycle in contrast to mathematical symmetry parameters for the whole gait cycle, like the cross-correlation coefficient describing the similarity of two curves [14].

The normalized symmetry index (SI_norm_, Eq. 2), similar to the SI but calculated with normalized data, was presented and validated by Gouwanda et al. [12, 14, 15]. Through a min–max normalization of the angular velocity, the complete curves are transformed, showing only positive values without zero-crossings, allowing the SI_norm_ to be calculated on each point of the gait cycle.

2 $${\text{SI}}_{{{\text{norm}}}} = \frac{{{\text{X}}_{{{\text{norm}}\left( {\text{R}} \right)}} - {\text{X}}_{{{\text{norm}}\left( {\text{L}} \right)}} }}{{0.5* \left( {{\text{X}}_{{{\text{norm}}\left( {\text{R}} \right)}} + {\text{X}}_{{{\text{norm}}\left( {\text{L}} \right)}} } \right)}}*100\% , \quad {\text{with}}\;{\text{X}}_{{{\text{norm}}\left( {\text{n}} \right)}} = \frac{{{\text{X}}_{{\text{n}}} - {\text{X}}_{\min } }}{{{\text{X}}_{\max } - {\text{X}}_{\min } }} + 1$$

Alternatively, a symmetry index (SI, Eq. 3) was presented by Nigg et al. as an integral over a complete gait cycle [9].

3 $${\text{SI}} = \mathop \smallint \limits_{1}^{100} {\text{SF}}\,{\text{dt}},\quad {\text{with}}\;{\text{SF}} = \frac{{\left| {{\text{X}}_{{\text{R}}} \left( {\text{t}} \right) - {\text{X}}_{{\text{L}}} \left( {\text{t}} \right)} \right|}}{{0.5*\left[ {{\text{range}}\left( {{\text{X}}_{{\text{R}}} \left( {\text{t}} \right)} \right) + {\text{range}}\left( {{\text{X}}_{{\text{L}}} \left( {\text{t}} \right)} \right)} \right]}}*100\%$$

In this method, the symmetry curve can be analyzed over the gait cycle using the symmetry function (SF) [9, 16].

Another normalized symmetry index (NSI, Eq. 4) was presented by Queen et al. [10].

4 $${\text{NSI}} = \frac{{{\text{X}}_{{{\text{R}}\left( {{\text{trial}}} \right)}} - {\text{X}}_{{{\text{L}}\left( {{\text{trail}}} \right)}} }}{{\max_{{{\text{trial}} = 1:{\text{n}}}} \left( {\max \left( {0,{\text{X}}_{{{\text{R}}\left( {{\text{trial}}} \right)}} , {\text{X}}_{{{\text{L}}\left( {{\text{trail}}} \right)}} } \right)} \right) - \min_{{{\text{trial}} = 1:{\text{n}}}} \left( {\min \left( {0,{\text{X}}_{{{\text{R}}\left( {{\text{trial}}} \right)}} , {\text{X}}_{{{\text{L}}\left( {{\text{trail}}} \right)}} } \right)} \right)}}*100\%$$

The calculation of the NSI requires the analysis of the individual measurement curves for each trail/ stride. In contrast to the analysis of each stride, usually in motion analysis, a mean curve of, e.g. the joint angle, is analyzed over one gait cycle [17]. While the NSI of Queen et al. was calculated only for single examined parameters [10], it can also be evaluated over a complete gait cycle due to the normalization.

This study aimed to identify the indices with the best diagnostic values related to kinematic data. To quantify symmetrical movements, the three identified and described symmetry indices were calculated, compared, and analyzed for different joint angles during walking, ascending, and descending stairs. While walking is a basic human movement, stair climbing is a more complex movement in everyday life with increased loads [18]. Therefore, increased asymmetry is assumed during stair climbing than while walking. Moreover, because of their connection over the lower limbs, comparable symmetrical movements of hip, knee, and ankle joint angles were hypothesized. Comparing different symmetry indices should help in the evaluation of the best method to analyze movement symmetry. Besides the comparative study, presenting reference values for symmetry indices of a normative, healthy test group can be helpful in further symmetry evaluations on diagnostics or rehabilitation.

## Material and methods

### Data collection

In a comparative pilot study, thirty-five healthy volunteers participated in the study and were analyzed during walking and stair climbing using an inertial measurement unit (IMU) system. The group consisted of 16 women and 19 men with an age of 26 ± 3 years and a body mass index (BMI) of 23 ± 2 kg/m^2^. Excluded were obese persons with a BMI > 35 kg/m^2^, persons with a leg length difference > 0.5 cm, or other diseases that can influence the movement behavior. Only participants without injuries and limitations of the musculoskeletal system were included, who were of legal age and able to understand the research project and give their (oral and written) consent. The study was approved by the local ethics committee (EC 278/19).

The IMU system MyoMotion (Noraxon U.S.A. Inc., Scottsdale, USA) was used for the motion analysis focused on the objective and dynamic detection of the joint angles. Seven IMUs in total were attached according to the manufacturer's specifications and were connected to each segment with the help of straps or clips. The sensors were placed at the following standardized locations: one centrally at the back of the pelvis over S1 with the x-axis pointing upwards, four at the front on the upper and lower legs, and two at the back of the feet. The leg sensors were positioned centrally from frontal view on the thigh and shinbone each below the main muscle belly, with the x-axis pointing upwards parallel to the leg axis. The participants were required to wear short sports pants and the same special therapeutic rehabilitation shoes (BORT therapeutic shoe comfort, Bort GmbH, Weinstadt-Benzbach, Germany), in an appropriate size and adjustment. Therefore, the sensors or straps could be fixed at the legs directly on the skin and by a clip on the defined position on the shoes above the cuneiforms with the x-axis pointing to the toes.

For each participant, after the sensor application, as the first step, a calibration was performed. For this, the participants stood in a place without magnetic interference on a step stool in a neutral position with extended knees and arms hanging sideways, which defines the zero-degree joint position. After a successful calibration, the movements of the participants were recorded, first while walking 7 m in a straight line. In a second step, participants walked 8 steps, with a width of 124 cm, a depth of 29 cm, and a height of 17 cm, up and downstairs, using a step-over-step technique. The steps link two resting places, on which the participants had the opportunity to turn around, rest and position adequately for the next task. Participants were instructed to begin ascending and descending the stairs by taking the first step with their right foot. The subjects had the opportunity of a test trial before the movements were recorded.

### Data processing

The data recorded at a sampling frequency of 100 Hz was stored and processed in the MyoResearch software (version MR 3.14, Noraxon U.S.A. Inc., Scottsdale, USA). The magnetic stabilization algorithm for the foot, shank, and thigh was used to reduce the influence of magnetic interference in the measurement environment, and the heavy anti-wobbling smoothing function was used to reduce soft tissue artifacts. For further processing and calculation of the symmetry indices, the measurement data was exported as MATLAB files. Based on a previously developed and presented algorithm [19], the individual strides were detected in the data and each stride was normalized/ interpolated to 100 points to be displayed over a gait cycle of 1–100% instead of over the time (in seconds) [20]. Strides were defined to start with the initial contact and end with the following initial contact of one foot, for walking and stair climbing in the same way. Based on the walking and stair climbing distances, 6 strides were detected and the average values over 3 left and right strides were calculated for each movement. Since the described 6 strides were not recorded/ detected in one data set, it was excluded from further analysis.

The main movements of the lower extremity during walking took place in the sagittal plane [16], therefore, the hip and knee flexion/extension, as well as the dorsi-/plantarflexion of the ankle joint were analyzed in this plane.

The symmetry parameters were calculated for all 100 points of the gait cycle and displayed as the symmetry progression over the gait cycle. The SI_norm_ was calculated based on Eq. 2. In addition to Gouwanda et al. who analyzed angle velocities [14], the SI_norm_ of the joint angles were calculated. Contrary to Gouwanda et al., the left and right joint angles were normalized based on their respective extrema instead of only by the extrema of the right joint angles to ensure only positive normalized values and no zero-crossing of the SI_norm_ [12, 14, 15]. SI and SF were calculated based on Eq. 3, using SF for the presentation of the symmetry throughout the gait cycle and SI for statistical evaluation. The NSI (Eq. 4) was calculated for each point of the gait cycle (1–100%) instead of only for the maximum joint angle [10]. The numerator represents the difference between the measured joint angle of the left and right side on one point of the gait cycle for a single stride. The denominator represents the maximum and minimum values for the particular measures across all three strides. The minimum is defined to be 0, in the case of only positive measures. The NSI is calculated for each stride and then averaged to get a symmetry score of all three strides. For statistical evaluation, the absolute maxima of the SI_norm_ and NSI over the entire gait cycle were determined.

### Statistical analysis

Statistical analysis was performed using IBM® SPSS Statistics software (IBM® SPSS Statistics v. 25, IBM Cooperation, Chicago, Illinois, USA) and a significance level of *p* = 0.05 was assumed. Exploratory data analysis (minimum, maximum, mean, and standard deviation of the different symmetry parameters) was used to present the reference values. Due to the data distribution, a log transformation (natural logarithm to the base of e) was performed on the data and subsequently transformed back for interpretation of the results, like the bias. The normal distribution of the (transformed) data was checked using Kolmogorov–Smirnov tests, Q–Q diagrams, and/or histograms.

The agreement of the 3 different symmetry indices was shown using Bland–Altman-Plots [21], and the significant bias was verified by paired t-tests. Based on the previously described guideline values of symmetry parameters for healthy subjects of up to ± 10% or 15% [11, 13, 15], a limit value of ± 5% was defined for the comparability.

Differences in the symmetry between the joint angles (hip, knee, ankle) and movements (walking, ascending, descending), as well as possible interaction effects of the two inner subject factors, were evaluated using a repeated measurement model (RM ANOVA). The sphericity of the data was checked in each case via the Mauchly test and, in case of violations, the Greenhouse–Geisser (GG, < 0.75) or Huynh–Feldt (HF, > 0.75) correction of the results was interpreted. Bonferroni correction was used for the post hoc pairwise comparison of two repeated conditions and a simple contrast analysis for the interactions.

## Results

**Fig. 1:**
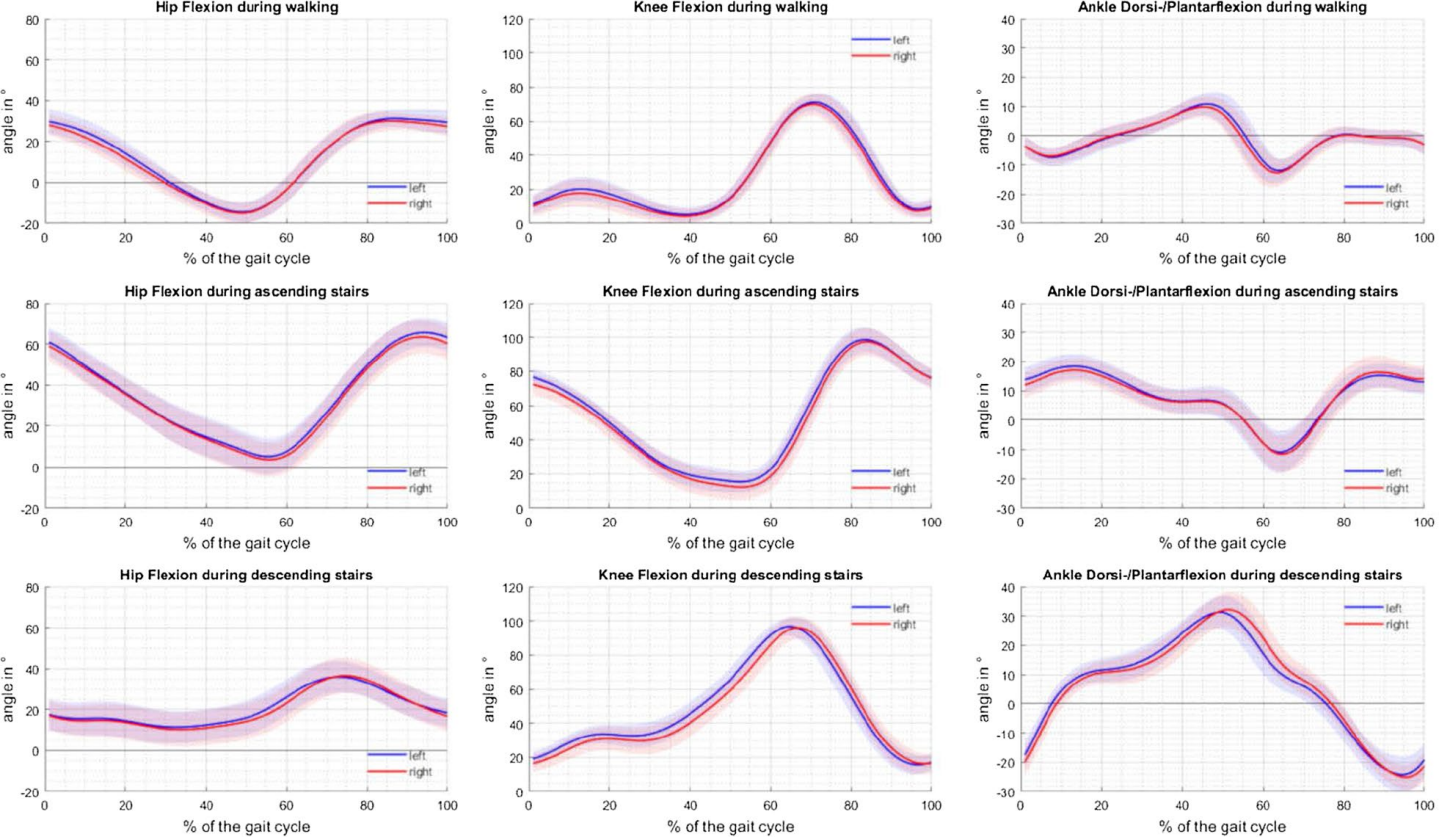
Joint angle progression. The joint angle progression over one gait cycle during walking (first row), ascending (second row), and descending stairs (third row) of the hip (first column), knee (second column), and ankle movement (third column) in the sagittal plane is shown

**Fig. 2:**
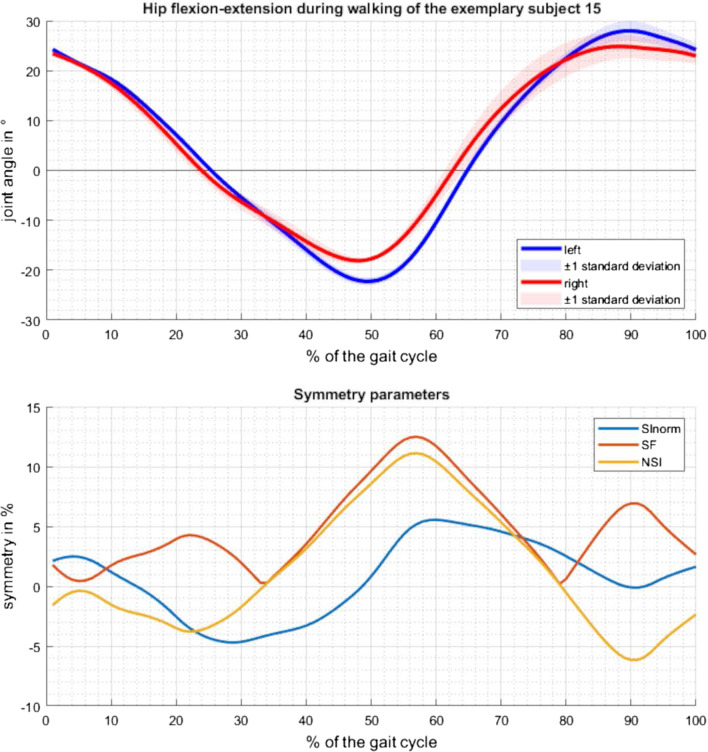
Joint angle and suitable symmetry parameters progression. Presentation of the joint angle progression for hip flexion–extension of the randomly selected participant 15 during walking and the suitable calculated symmetry parameters

The joint angle progressions over one gait cycle as mean curves over all participants were presented as the basis (Fig. 1). Descriptive statistics of the symmetry parameters are presented in Table 1. In Fig. 2, the hip flexion–extension progression in comparison with the three different symmetry parameter curves is presented for the randomly selected participant 15 during walking. In the appendix (Fig. 4), the symmetry parameter curves for participant 15 for all joint angles and movements were presented.

**Table 1:** Descriptive statistics of the symmetry parameters of the N = 34 participants as reference values

Symmetry parameter	Joint	Movement	Minimum	Maximum	Mean	Standard deviation
maximum SI_norm_ in %	Hip	Walking	2.10	13.59	6.77	3.07
		Ascending stairs	1.90	15.97	5.74	2.95
		Descending stairs	5.48	36.92	14.50	7.50
	Knee	Walking	2.45	19.82	8.67	4.70
		Ascending stairs	2.19	18.49	6.76	3.33
		Descending stairs	3.46	30.70	10.42	5.77
	Ankle	Walking	7.91	35.99	16.31	6.10
		Ascending stairs	3.60	28.50	12.03	5.81
		Descending stairs	1.72	28.22	8.85	4.55
SI in %	Hip	Walking	1.54	17.69	6.81	3.29
		Ascending stairs	1.14	15.70	5.94	3.11
		Descending stairs	4.36	29.51	12.40	5.65
	Knee	Walking	1.73	17.95	6.71	4.19
		Ascending stairs	2.46	17.91	6.66	3.77
		Descending stairs	1.90	21.13	8.00	4.10
	Ankle	Walking	3.92	39.67	12.19	8.08
		Ascending stairs	4.67	23.31	10.63	5.23
		Descending stairs	2.09	18.42	7.03	3.25
maximum NSI in %	Hip	Walking	3.64	20.32	11.97	4.21
		Ascending stairs	2.63	18.81	9.83	3.86
		Descending stairs	6.02	38.31	17.76	8.24
	Knee	Walking	3.57	30.85	13.46	8.04
		Ascending stairs	4.55	26.55	11.20	4.61
		Descending stairs	3.97	35.51	14.77	7.40
	Ankle	Walking	8.76	49.94	22.62	10.45
		Ascending stairs	7.50	42.09	20.71	8.58
		Descending stairs	4.70	44.14	15.28	7.56

**Fig. 3:**
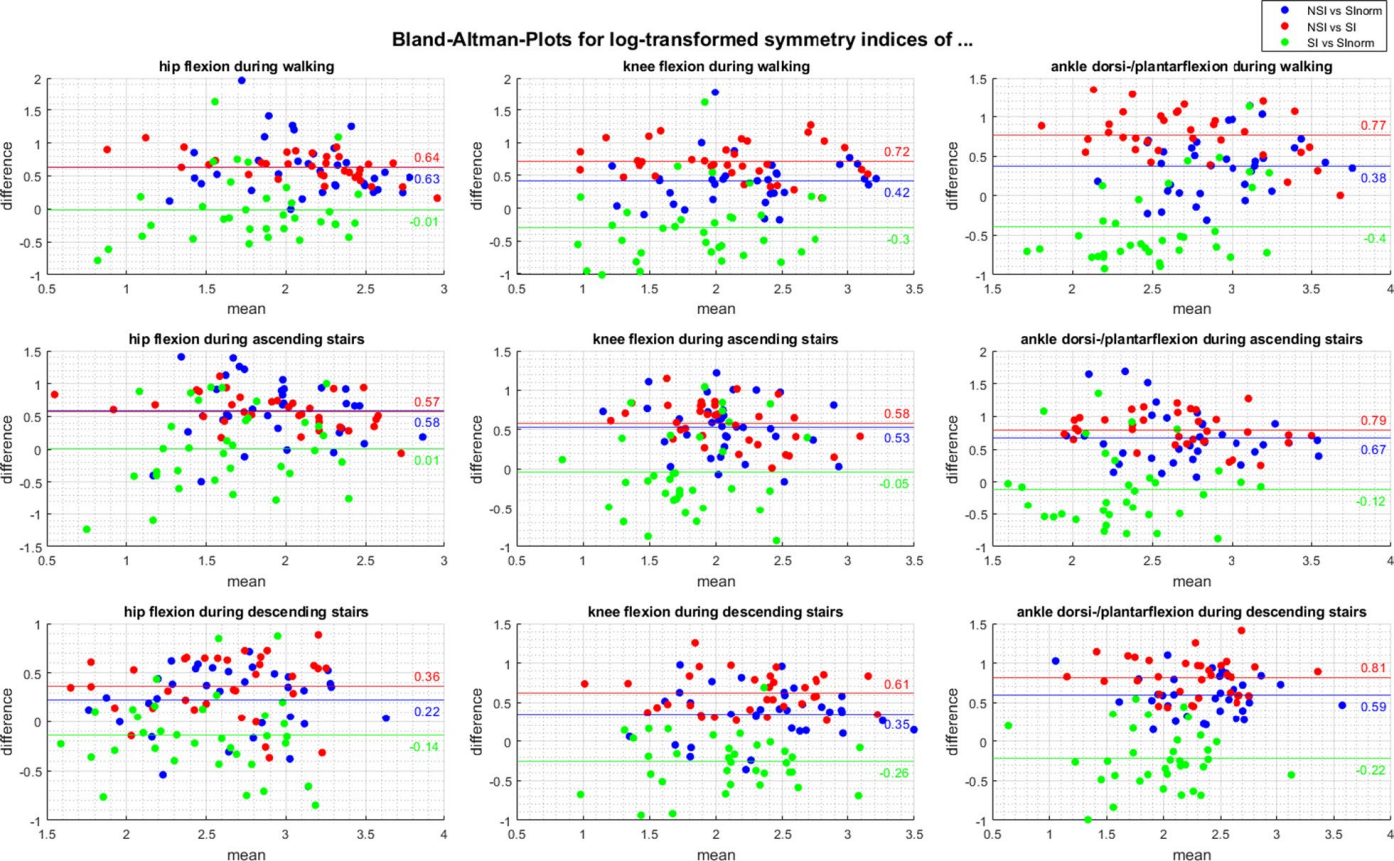
Bland–Altman-Plots for comparison of the 3 different symmetry parameters NSI, SI_norm_, and SI. The log-transformed symmetry indices of the joint angles during walking (first row), ascending (second row), and descending stairs (third row) of the hip (first column), knee (second column), and ankle movement (third column) in the sagittal plane are shown

**Fig. 4:**
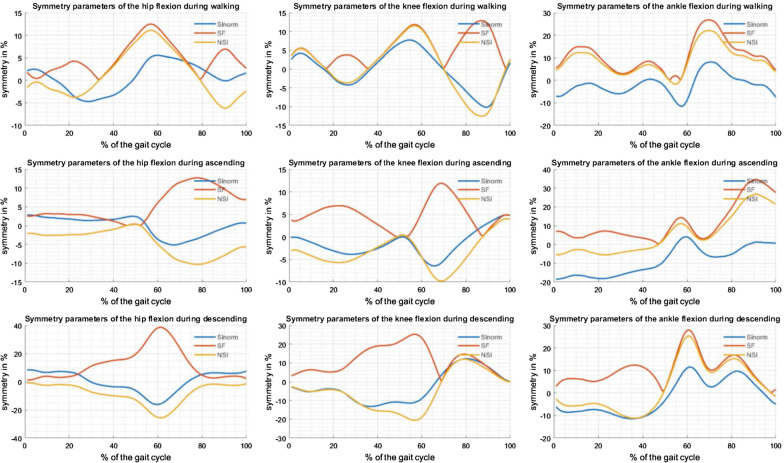
Symmetry parameter curves over one gait cycle. Presentation of the symmetry parameter curves of participant 15 during walking (first row), ascending stairs (second row), and descending stairs (third row) of the hip (first column), knee (second column), and ankle movement (third column) in the sagittal plane

To compare the different symmetry parameters, the Bland–Altman-Plots are shown in Fig. 3. The plots show the difference of 2 log-transformed symmetry parameters versus their mean value. The bias was defined as the mean difference between two parameters after back transformation with limits of agreement (LoA) as the mean difference plus and minus 2 standard deviations (Table 2). In most cases, the bias deviates significantly from zero (*p* < 0.001, Table 2). The difference between the SI and SI_norm_ is small for the hip flexion and the knee flexion during ascending stairs resulting in no significant difference (*p* ≥ 0.05, Table 2). However, the LoA is below the predefined limits of acceptance of 5% for all cases.

**Table 2:** Back transformed bias with limits of agreement for the comparison of the symmetry indices

Joint angle during movement	Comparison	Bias in %	Lower limits of agreement in %	Upper limits of agreement in %	T-Test (*p* value)
Hip flexion during walking	NSI versus SI_norm_	1.87	0.81	4.31	< 0.001
	NSI versus SI	1.90	1.28	2.82	< 0.001
	SI versus SI_norm_	0.99	0.36	2.69	0.888
Hip flexion during ascending stairs	NSI versus SI_norm_	1.79	0.70	4.56	< 0.001
	NSI versus SI	1.77	1.07	2.95	< 0.001
	SI versus SI_norm_	1.01	0.31	3.30	0.953
Hip flexion during descending stairs	NSI versus SI_norm_	1.25	0.64	2.45	0.001
	NSI versus SI	1.44	0.77	2.69	< 0.001
	SI versus SI_norm_	0.87	0.40	1.90	0.050
Knee flexion during walking	NSI versus SI_norm_	1.52	0.73	3.17	< 0.001
	NSI versus SI	2.05	1.19	3.55	< 0.001
	SI versus SI_norm_	0.74	0.26	2.15	0.003
Knee flexion during ascending stairs	NSI versus SI_norm_	1.71	0.86	3.38	< 0.001
	NSI versus SI	1.79	1.04	3.07	< 0.001
	SI versus SI_norm_	0.95	0.34	2.69	0.613
Knee flexion during descending stairs	NSI versus SI_norm_	1.42	0.76	2.68	< 0.001
	NSI versus SI	1.85	1.14	3.01	< 0.001
	SI versus SI_norm_	0.77	0.40	1.50	< 0.001
Ankle dorsi-/plantarflexion during walking	NSI versus SI_norm_	1.46	0.71	3.00	< 0.001
	NSI versus SI	2.17	1.18	3.97	< 0.001
	SI versus SI_norm_	0.67	0.26	1.77	< 0.001
Ankle dorsi-/plantarflexion during ascending stairs	NSI versus SI_norm_	1.96	0.89	4.32	< 0.001
	NSI versus SI	2.21	1.34	3.66	< 0.001
	SI versus SI_norm_	0.89	0.29	2.70	0.022
Ankle dorsi-/plantarflexion during descending stairs	NSI versus SI_norm_	1.81	1.11	2.94	< 0.001
	NSI versus SI	2.26	1.39	3.66	< 0.001
	SI versus SI_norm_	0.80	0.39	1.66	0.001

The statistical models show that for all 3 parameters there is a significant difference in the symmetry values of the different joint angles (*p* < 0.001, Table 3).

**Table 3:** Statistical parameter of the repeated measurement models

Symmetry parameter		Maximum SI_norm_	SI	Maximum NSI
*Main effect (p, η*^*2*^*)*	Joint	< 0.001, 55%	< 0.001 (HF), 28.5%	< 0.001, 57.1%
	Movement	0.001, 19.2%	0.055, 8.4%	0.140, 5.8%
	Interaction	< 0.001 (HF), 56.3%	< 0.001, 59%	< 0.001, 46.6%
*Post Hoc analysis joint (p)*	Hip versus knee	1.000	0.039	1.000
	Hip versus ankle	< 0.001	0.056	< 0.001
	Ankle versus knee	< 0.001	< 0.001	< 0.001
*Post Hoc analysis movement (p)*	Walking versus ascending stairs	0.018	0.981	0.196
	Walking versus descending stairs	0.881	0.500	1.000
	Ascending versus descending stairs	0.003	0.070	0.250
*Contrast analysis interaction (p)*	Hip versus knee and walking versus ascending stairs	0.651	0.037	0.209
	Hip versus knee and walking versus descending stairs	< 0.001	< 0.001	0.008
	Hip versus ankle and walking versus ascending stairs	0.183	0.541	0.247
	Hip versus ankle and walking versus descending stairs	< 0.001	< 0.001	< 0.001

In the comparison of the three joints, the knee movement (in the sagittal plane) shows the lowest values and thus the highest symmetry, while the ankle joint shows the greatest asymmetry. The symmetry values of the different movements are significantly different only for the SI_norm_ (SI_norm_ *p* = 0.001, SI *p* = 0.055, NSI *p* = 0.202, Table 3). The greatest symmetry was not seen during walking but during ascending stairs, while the greatest asymmetry was seen while descending stairs. The interaction terms of the two factors (joint and movement) are significantly different for all symmetry parameters (*p* < 0.001, Table 3). While the hip and knee show a greater symmetry during walking and ascending than descending stairs, this effect is reversed for the ankle joint with the greatest asymmetry during walking and the greatest symmetry during descending.

## Discussion

To quantify the differences and pointing out a leading discrete value describing a right/left asymmetry for dynamic joint angles is a common clinical and research objective. The purpose of this work was a comparison of the most common symmetry computations to achieve a recommendation for standardized practice.

The reference values for the symmetry of the lower limb movements in the sagittal plane during walking and stair climbing were presented, compared, and analyzed based on three different symmetry parameters (SI_norm_, SI, and NSI). Contrary to our hypotheses, but comparable with existing literature, the symmetry showed significant differences between the joints, underlining the need for independent symmetry analysis of the lower limb joint angles despite their link [9, 16]. Moreover, the differences between walking and stair climbing were only significant based on the SI_norm_ calculations. The impact of a specific movement on joint angle symmetry was more important for the interaction with the joints, as the interaction effect was shown to be significant. The ranking of joint symmetry changes between different movements.

The comparison of SI_norm_, SI, and NSI showed good agreement between these parameters for all analyzed dynamic joint angles, as the LoA were below the predefined limit of 5%. Nevertheless, as a significant bias was found in most comparisons, shifted values have to be assumed. Analyzing joint angles, the highest symmetry values were calculated by the NSI, followed by the SI_norm_ and SI, but the widest range of the LoA was calculated between the NSI and the SI_norm_. Explaining the smaller range between the SI_norm_ and the SI with the small bias, a slightly different progression of the SI_norm_ can be supposed. Thus, only the significant movement effect in the statistical model of the SI_norm_ can be explained.

The SI_norm_, introduced and validated in 2011 by Gouwanda et al. is a bounded parameter (0–100%) for symmetry calculations [12, 14, 15]. For healthy individuals, the SI_norm_ was presented to be in a range of ± 15% for thigh and shank angular rate [12, 14, 15]. No previous data exist for other gait parameters like joint angles or of real patients. Nevertheless, compared to the limits of ± 15% for angular rates [12, 14, 15], mean SI_norm_-values up to 16% were found for the joint angles symmetry (Table 1). The individual analysis of the SI_norm_ showed increased values in some participants with values of up to 37% (Table 1). As the standard deviation for the SI_norm_ was in a range of 2.95% up to 7.50%, these values could be defined as outliers. Nevertheless, it underlines the importance of a careful interpretation of symmetry values, since increased asymmetric movements can also occur in healthy participants for various reasons.

The SI showed a similar distribution of individual symmetry values with outliers up to 40% (Table 1). Compared to the SI_norm_, the range of 3.11–8.08% for the standard deviations of the SI was increased, but with decreased variations during descending stairs (Table 1). In contrast to the SI_norm_, with the analysis of the curve’s maxima, the integral of the SF was calculated [9]. Therefore, also variations over the gait cycle apart from the maxima were considered, possibly responsible for the differences between SI and SI_norm_. A similar variation of the SI between the analyzed participants was also previously described [9, 16, 22]. Contrary to the SI_norm_, the SI with the SF, introduced by Nigg et al. [9], was also used in subsequent studies by different researchers [16, 22, 23], for example, to analyze the symmetry differences between barefoot walking and walking with two different shoes [16]. Compared to the previously presented SI values for walking with normal shoes, we found increased symmetry of the knee angle and asymmetry of ankle dorsi-/plantarflexion [16]. Possible explanations are our analysis of the whole gait cycle instead of the stance phase, and the use of an IMU system instead of an optical motion capture system [9, 16, 22]. Less accurate detection of the ankle movement, with an IMU system, is possible because of magnetic interference from electric cables under the floor and/or a weak sensor fixation on the shoes.

Higher SI values of the hip, clearly higher SI values of the knee, and lower SI values of the ankle movement described by Nigg et al. [9], can be explained by the joint angles being analyzed during running. Compared to the presented significant interaction effects of the joint and movement factors, during running a changed ranking of the symmetry values for the specific joints, can be assumed. In addition to the symmetry analysis of joint angles, the SI was also used to analyze joint moment symmetry [22], underlining the manifold use of the SI and SF.

Besides the use of the SI_norm_ and the SI for the symmetry analysis of two different measurement parameters, the NSI was developed as a universal symmetry index for a wide range of measurement parameters [10]. The NSI as a bounded parameter (0–100%), introduced by Queen et al. [10], was the newest parameter presented in this study. Therefore, no reference values for the NSI were found in previous studies while we also expanded the NSI. The NSI was introduced to be calculated from a single parameter, like the peak knee valgus angle [10], instead of the knee motion over one gait cycle. Compared to the SI_norm_, we calculated the NSI for each point of the gait cycle and used the absolute maximum over the gait cycle for statistical evaluation. However, the NSI showed a high variance compared to the other symmetry parameters. One reason for this could be that the calculation of NSI was based on the data from multiple rather than single trials [10] or in other words, the calculation of NSI was based on the angle curves of every stride instead of a mean angle progression over all strides.

There exist some limitations. As one of the first pilot comparison studies, we analyzed only a small number of participants with a specific range of age. Therefore, new reference values for the symmetry parameters may be necessary for the comparison of patients of other ages. Additionally, as our study analyzed the joint angles of the lower limbs in the sagittal plane, analyzing different movement planes, joint angles, measurement parameters, and movements will contribute to a better and holistic understanding of the symmetry of human motion. Moreover, only three different methods were chosen, improved, and presented. The analysis of a mean curve instead of analyzing raw data decrease the statistical power, but the presence of measurement data over a gait cycle is a commonly used method for data reduction, interpretation, and comparison [17], and decreases the impact of extreme values caused by uncommon movements or measurement errors. There exist much more methods to quantify symmetrical movements, but the presented methods, comparisons, and reference values are the first step on the way to a better understanding and the most suitable method.

## Conclusion and outlook

As shown in this paper, there are several functions in the literature which describe symmetry; however, all are slightly different. The presented methods to quantify movement symmetry are comparable, apart from a bias. Analyzing dynamic joint angle symmetry of healthy participants during walking and stair climbing significant differences between hip, knee, and ankle (dorsi-/plantar) flexion were noted and have to be taken into account. Moreover, the specific joint angle symmetry changes significantly between different movements. The presented methods, results, and reference values are a necessary and helpful first step for further symmetry analysis, nevertheless, if it is for a deeper biomechanical insight, a diagnostic tool to identify pathological movements or to track a rehabilitation process.

## Data Availability

The datasets used and/or analyzed during the current study are available from the corresponding author on reasonable request.

## Appendix

See Fig. 4.

## Abbreviations

SI: Symmetry index (Eq. 1)
SI_norm_: Normalized symmetry index introduced by Gouwanda et al. (Eq. 2)
SI: Symmetry index introduced by Nigg et al. (Eq. 3)
SF: Symmetry function introduced by Nigg et al. (Eq. 3)
NSI: Normalized symmetry index introduced by Queen et al. (Eq. 4)
IMU: Inertial measurement unit
RM ANOVA: Repeated measurement analysis of variance
GG: Greenhouse–Geisser
HF: Huynh–Feldt
LoA: Limits of agreement
